# Function and quality-of-life of survivors of pelvic and lower extremity osteosarcoma and Ewing's sarcoma: the Childhood Cancer Survivor Study

**DOI:** 10.1038/sj.bjc.6602220

**Published:** 2004-11-09

**Authors:** R Nagarajan, D R Clohisy, J P Neglia, Y Yasui, P A Mitby, C Sklar, J Z Finklestein, M Greenberg, G H Reaman, L Zeltzer, L L Robison

**Affiliations:** 1 Division of Pediatrics, Epidemiology and Clinical Research, Department of Pediatrics, University of Minnesota, Mayo Mail Code 484, 420 Delaware St., SE Minneapolis, MN 55455, USA; 2 Department of Orthopedic Surgery, University of Minnesota, Mayo Mail Code 806, 420 Delaware St., SE Minneapolis, MN 55455, USA; 3 Division of Hematology/Oncology/Blood and Marrow Transplant, Department of Pediatrics, University of Minnesota, Mayo Mail Code 484, 420 Delaware St, SE Minneapolis MN 55455, USA; 4 Cancer Prevention Research Program. Fred Hutchinson Cancer Research Center, 1100 Fairview Ave. N., PO Box 19024 Seattle, WA, 98109, USA; 5 Department of Pediatrics, Memorial Sloan Kettering Cancer Center, 1275 York Ave, New York, NY 10021, USA; 6 Department of Pediatric Hematology/Oncology, Jonathan Jacques Childrens Cancers Cancer Center, 2653 Elm Ave, Suite 200, Long Beach, CA 90806, USA; 7 Department of Hematology/Oncology, Hospital for Sick Children, 555 University Ave., Toronto, Ontario M5G1X8, USA; 8 Department of Hematology/Oncology, Children's National Medical Center, 111 Michigan Ave, NW, Washington DC 20010, USA; 9 Department of Pediatrics, University of California at Los Angels, 10833 Le Conte Ave., #22-464 MDCC, Los Angeles, CA 90095, USA

**Keywords:** osteosarcoma, Ewing's sarcoma, quality of life, function, lower extremity, bone tumour

## Abstract

Limb-sparing surgeries have been performed more frequently than amputation based on the belief that limb-sparing surgeries provide improved function and quality-of-life (QOL). However, this has not been extensively studied in the paediatric population, which has unique characteristics that have implications for function and QOL. Using the Childhood Cancer Survivor Study, 528 adult long-term survivors of pediatric lower extremity bone tumours, diagnosed between 1970 and 1986, were contacted and completed questionnaries assessing function and QOL. Survivors were an average of 21 years from diagnosis with an average age of 35 years. Overall they reported excellent function and QOL. Compared to those who had a limb-sparing procedure, amputees were not more likely to have lower function and QOL scores and self-perception of disability included general health status, lower educational attainment, older age and female gender. Findings from this study suggest that, over time, amputees do as well as those who underwent limb-sparing surgeries between 1970 and 1986. However, female gender, lower educational attainment and older current age appear to influence function, QOL and disability.

Malignant bone tumours, predominantly osteosarcoma and Ewing's sarcoma, account for approximately 6% of all cancer diagnosed under the age of 20 years (Ries *et al*, 1999) in the United States. Nearly two-thirds of these tumours have a primary site in the lower extremity of pelvis. Historically, treatment of extremity bone tumours with amputation resulted in poor survival rates (Wang and Schulz, 1953; Dahlin *et al*, 1961; Friedman and Carter, 1972). Beginning in the 1970s, treatment strategies changed to include the use of multiagent chemotherapy (Rosen *et al*, 1979; Link *et al*, 1986; Nesbit *et al*, 1990). This led to significant improvements in the prognosis of children and young adults with osteosarcoma and Ewing's sarcoma and increased the overall survival rate for patients with nonmetastatic disease from 10 to 20% (Wang and Schulz, 1953; Dahlin *et al*, 1961; Friedman and Carter, 1972) to approximately 60% (Burgert *et al*, 1990; Bacci *et al*, 1998; Nesbit *et al*, 1990; Provisor *et al*, 1997). Enabled by improved radiographic and surgical techniques, the surgical management of patients with lower extremity lesions was increasingly characterized by an expanded use of limb-sparing surgeries (Springfield, 1991).

While there is general agreement that limb-sparing techniques are the preferred approach for patients with upper extremity tumours (Cheng and Gebhardt, 1991; Aboulafia and Malawer, 1993), debate continues regarding the long term outcomes of amputation compared to limb-sparing surgery for lower extremity primaries (Nagarajan *et al*, 2002). The current investigation assessed self-reported function and quality of life (QOL) among a large cohort of long-term survivors of childhood lower extremity bone tumours.

## MATERIALS AND METHODS

### Subject selection and eligibility

The childhood Cancer Survivor Study (CCSS) is a cohort of individuals with a confirmed diagonosis of cancer who participated in the Long-Term Follow-Up Study, a multi-institutional study of individuals who survived for 5 or more years after treatment for cancer, leukaemia, tumours or similar illnesses diagnosed during childhood or adolescence. The methods and cohort characteristics of the CCSS have been previously presented in detail (Robison *et al*, 2002). Briefly, as of 1 November 2000, 20 276 participants met the cohort inclusion criteria of: (1) diagnosis of one of the following cancers: brain tumour, leukaemia, Hodgkin's disease, non-Hodgkin's lymphoma, kidney cancer, neuroblastoma, soft-tissue sarcoma, or cancer of the bone; (2) initial treatment of one of the 25 collaborating CCSS institution – see Appendix; (3) diagnosis date between 1 January 1970 and 31 December 1986; (4) age less than 21 years at diagnosis; and (5) survival 5 years from diagnosis. A total of 1042 of the 1596 5-year survivors of a bone tumour directly participated by completing the CCSS baseline data collection questionnaire. Excluded were 328 survivors who declined participation, 221 considered lost to follow-up after extensive tracing, and five who were pending. The present report is restricted to the subset of 629 participants who fulfilled the following additional criteria: (1) diagnosis of osteosarcoma or Ewing's sarcoma: (2) tumour located in the lower extremity or the pelvis: (3) 18 years of age or older at the time of the present evaluation: (4) availability of complete medical records: (5) alive and able to complete a self-reported (not by proxy) function and QOL assessment.

Overall, 84% (528/629) of the subjects contacted for the current study participated by completing the function and QOL questionnaire. Of the 101 nonparticipants, two were lost to follow-up, 75 declined participation and 24 were pending participation at the time of analysis. Nonparticipants were slightly more likely than participants to be younger at diagnosis (12.7 years *vs* 13.5 years) and to have an extremity lesion (97 *vs* 91%), but were less likely to have graduated from college (27 *vs* 48%).

The CCSS protocol and contact documents were reviewed and approved by the Human Subjects Committee at each participating institution. All contact documents including the baseline questionnaire and the function and QOL assessments can be viewed at www.cancer.umn.edu/ccss.

### Assessment of function and QOL

Subjects completed a self-administered questionnaire that included the Toronto Extremity Salvage Score (TESS) (Davis *et al*, 1996, 1999) and the Quality of Life for Cancer Survivors (QOL-CS) instrument (Ferrel *et al*, 1995a, 1995b) The TESS (30 questions) measures physical disability based on the patient's report of their function and was developed and validated specifically for individuals who have undergone surgery for lower extremity musculoskeletal tumours. The QOL-CS questionnaire (41 questions), developed and validated specifically for cancer survivors, measures four domains of QOL (physical, psychological, social, and spiritual well-being).

### Cancer treatment information

Information on the characteristics of the original cancer diagnosis was obtained on all consented eligible cases from the treating institution. Surgical local control procedures were grouped into amputation and nonamputation by using the abstracted ICD-9 codes and participants' questionnaire responses. Subjects (*n*=24) with an amputation 3 or more years after diagnosis were classified by their initial surgical treatment.

Site codes (long bones of the lower extremity or pelvis) from the CCSS medical records abstraction forms were the primary source for classifying tumour site. More specific site information (e.g. distal femur or proximal tibia) was obtained using a skeletal diagram given to the bone tumour survivors, who were asked to indicate the site of their tumour.

### Data analysis

*A priori* participants were categorized to one of four groups defined by age at diagnosis of the bone tumour (⩽12 years *vs* ⩾12 years) and by the type of surgery (amputation *vs* limb-sparing surgery) resulting in four age/surgery groups (⩽12/Amp and ⩾12/Amp: under and over the age of 12 years with an amputation; ⩽12/LS and ⩾12/LS: under and over the age of 12 years with a limb-sparing surgery). For comparison purposes, the ⩾12/LS age-surgery group was used as the reference group. The age cutoff of 12 years old was chosen because it represents an approximate marker for emotional maturity (preadolescence and early adolescence) (Brindis *et al*, 1992) and pubertal development, and is the approximate age at which significant bone growth starts (Kreipe, 1992). Amputees were further subdivided into amputation only and amputation with radiation therapy. Nonamputees (limb-salvage) were categorised into: (1) radiation treatment only, (2) arthrodesis, (3) arthrodesis with radiation treatment, (4) endoprosthetic reconstruction, (5) endoprosthetic reconstruction with radiation treatment, (6) surgery not otherwise specified and (7) surgery not otherwise specified with radiation treatment. There were no rotationplasties noted. General health status was assessed from questions from the initial baseline survey of CCSS participants that asked about self-perceived general health. Responses were dichotomised into good/very good/excellent health *vs* fair/poor health.

TESS and QOL-CS questionnaires were scored according to instructions of the authors of the respective scales. TESS and QOL-CS scores were dichotomised for analyses by using the 25th percentile of the scores. Comparisons of TESS and QOL-CS scores across the age/surgery groups were performed using general linear models, adjusting for perceived general health, gender, educational attainment and age at the completion of the CCSS baseline questionnaire. The TESS scale incorporated a self-rating scale of disability, which was collapsed into a dichotomous variable: not disabled (not at all disabled and mildly disabled) and disabled (moderately and completely/severely disabled). The associations of the age/surgery variable with the dichotomous disability variable and with scoring below the 25th percentile on TESS or QOL-CS were assessed using logistic regression models, adjusting for general health, gender, educational attainment, and a categorical age at questionnaire completion variable. In order to assure that adjustment for general health status did not influence the relationship between the age/surgery groups and the outcomes, multivariate analyses were conducted with and without inclusion of the general health status variable, which provided comparable results. Given that the primary comparisons are based upon testing of *a priori* hypotheses, no correction was made for multiple comparisons.

## RESULTS

### Characteristics of study participants

In total, 80% of the participants had a previous diagnosis of osteosarcoma. The most frequent site of tumour was the distal femur (42%), followed by the proximal tibia (18%) (see Table 1

**Table 1:** Characteristics of study population

**Characteristic**	**Lower extremity survivors (*n*=528)**
*Gender*	
Female	270 (51.1%)
Male	258 (48.9%)
	
*General health*	
Poor	49 (9.7%)
Good	457 (90.3%)
	
*Education*	
No high school	21 (4.2%)
High school graduate	235 (47.5%)
College graduate	239 (48.3%)
	
*Specific site*	
Distal femur	226 (42.8%)
Proximal tibia	98 (18.6%)
Pelvis	46 (8.7%)
Other femoral sites	63 (11.9%)
Other tibial sites	46 (8.7%)
Fibular sites	39 (7.4%)
Nonspecific, nonpelvic site	10 (1.9%)
	
*Tumor type*	
Osteosarcoma	422 (79.9%)
Ewing's sarcoma	106 (20.1%)
	
*Surgery type*	
Amputation	
Amputation only	317 (60.0%)
Amputation/XRT	19 (3.6%)
Limb-sparing	
XRT only	32 (6.1%)
Arthrodesis/XRT	1 (0.2%)
Endoprosthesis/XRT	7 (1.3%)
NOS/XRT	40 (7.6%)
Arthrodesis	10 (1.9%)
Endoprosthesis	49 (9.3%)
Nonamputation, nonspecific	53 (10.0%)
	
*Age at diagnosis*	
Mean (range)	13.5 (1–20)
Median	14.0
	
*Years from diagnosis to questionnaire completion*	
Mean (range)	20.8 (13–31)
Median	21.0
	
*Age at questionnaire completion (years)*	
Mean (range)	34.8 (19.49)
Median	35.0
⩽30	132 (25.0%)
31–35	145 (27.5%)
36–39	128 (24.2%)
⩾40	123 (23.3%)

). The majority of participants had undergone amputation (63) and was over the age of 12 years at diagnosis (65%). Figure 1

**Figure 1:**
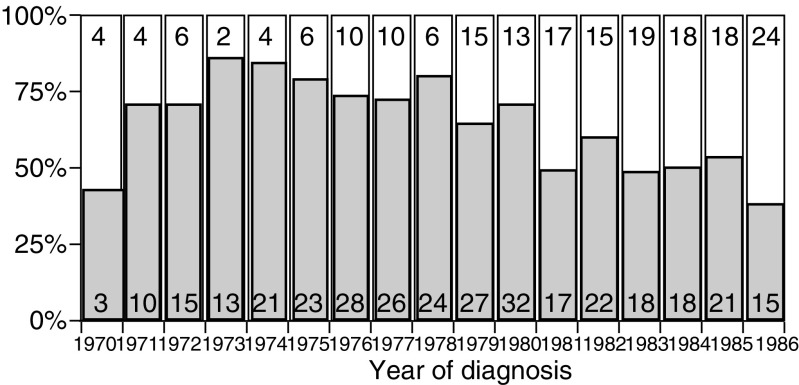
Frequency of amputation over time. Shaded bars represent the number of amputations and open bars represent the number of limb sparing procedures.

graphically represents the number of amputations performed by year that appears to decrease with time. The median time between diagnosis and evaluation was 21 years (range 12–31 years). The majority of survivors reported an educational level beyond high school and considered themselves to be in good health.

### QOL, function and self-perception of disability (Tables 2, 3 and 4)

Subjects in all four age/surgery groups scored relatively high with mean function scores (scale of 1–100) between 83 and 88 and mean quality-of-life scores (scale of 1–10) between 6.8. and 7.0 (Table 2

**Table 2:** Raw scores of function (TESS), quality of life (QOL-CS), quality of life subscales and percent self-reported as disabled

		**Age/surgery subgroups**			
**Outcome**	**All participants (*n*=528)**	**⩽12/Amp (*n*=121)**	**⩽12/LS (*n*=63)**	**>12/Amp (*n*=215)**	**>12/LS (*n*=129)**
*TESS*					
Mean (SD)	85.5 (14.3)	87.6 (12.4)	88.6 (15.7)	83.8 (13.1)	84.5 (16.8)
Range	17.2–100	64.2–100	23.3–100	33.7–100	17.2–100
*QOL-CS*					
Overall					
Mean (SD)	6.9 (1.4)	7.0 (1.4)	7.0 (1.2)	6.8 (1.3)	6.8 (1.4)
Range	1.4–9.7	6.3–9.4	3.4–9.0	3.2–9.7	2.4–8.9
Spirituality					
Mean (SD)	6.0 (2.0)	5.6 (19)	6.3 (1.8)	6.0 (2.0)	6.1 (1.9)
Range	0.1–10	0.1–10	2.3–10	0.7–10	1.3–9.7
Social					
Mean (SD)	7.4 (1.9)	7.5 (1.9)	7.6 (2.0)	7.3 (1.9)	7.4 (2.0)
Range	0.9–10	0.9–10	1.0–10	1.4–10	1.0–10.0
Psychosocial					
Mean (SD)	6.5 (1.6)	6.8 (1.6)	6.5 (1.5)	6.4 (1.6)	6.4 (1.6)
Range	0.9–9.9	0.9–9.9	2.7–9.2	2.2–9.8	2.0–9.3
Physical					
Mean (SD)	7.9 (1.6)	8.2 (1.6)	8.2 (1.5)	7.9 (1.7)	7.7 (1.6)
Range	1.0–10	1.9–10	4.0–10	1.0–10	1.6–10.0
					
*Self-rating disability (expressed as subjects (%))*					
Severely/completely	24 (4.6%)	2 (1.7%)	3 (4.8%)	11 (5.1%)	8 (6.3%)
Moderately	110 (21.0%)	27 (22.5%)	8 (12.7%)	54 (25.4%)	21 (16.4%)
Mildly	241 (46.0%)	60 (50.0%)	22 (34.9%)	112 (52.6%)	47 (36.7%)
Not disabled	149 (28.4)	31 (25.8%)	30 (47.6%)	36 (16.9%)	52 (40.6%)

) with no statistical difference in scores across groups. TESS and QOL-CS scores correlated with self-report assessments of disability, having Pearson's, correlation coefficients of 0.62 and 0.45, respectively. Mean TESS scores for self-ratings of completely/severely disabled, moderately disabled, mildly disabled, and not at all disabled were 61.2, 74.4, 86.9, and 95.4, respectively. Mean QOL-CS scores for these same groups were 5.0, 6.2, 7.0, and 7.6. When the self-rating scores were collapsed into those who considered themselves disabled or not disabled, significant differences were found between the two disability groups in mean TESS (72.1 *vs* 90.1, *P*⩽0.001) and QOL-CS scores (6.0 *vs* 7.2, *P*⩽0.001).

Characteristics of the survivors classified as disabled or scoring below the 25th percentile of TESS and QOL-CS are provided in Table 3

**Table 3:** Characteristics of survivors reporting disability and TESS and QOL-CS scores below the 25th

**Characteristic**	**No.**	**Self-rating disabled (%)**	**TESS score below 25th percentile (%)**	**QOL-CS score below 25th percentile (%)**
*Age/surgery group*				
⩽12/Amp	121	24.2	15.8	20.0
⩽12/LS	63	17.5	17.5	22.2
>12/Amp	215	30.5	28.6	29.1
>12/LS	129	22.7	28.1	25.8
				
*Amputation status*				
Amputation	336	28.2	24.0	25.8
Limb-sparing surgery	192	20.9	24.6	24.6
				
*General health*				
Good/excellent health	457	27.7	21.9	21.4
Fair/poor health	49	61.2	53.1	69.4
				
*Gender*				
Female	270	26.2	29.2	28.8
Male	258	24.9	19.1	21.8
				
*Education*				
No H.S. graduation	21	57.1	52.4	47.6
H.S. graduation	235	27.4	23.9	23.5
College graduation	239	21.1	21.1	25.3
				
*Age at questionnaire completion*				
⩽30 years old	132	16.8	15.3	17.6
31–35 years old	145	22.9	22.8	22.9
36–39 years old	128	26.0	26.8	30.7
⩾40 years old	123	27.7	32.8	31.2

. In pairwise comparisons of the age/surgery groups, no significant differences in mean TESS or QOL-CS scores, self-perception of disability, or scoring below the 25th percentile on QOL-CS or TESS scores were found after adjusting for general health, gender, educational status, and age at questionnaire completion (data not shown).

Poor general health status, older current age, and failure to graduate from high school predicted self-perception of disability and lower TESS and QOL-CS scores and were all statistically significant in univariate analyses (Table 4

**Table 4:** Odds ratio for considering themselves disabled (moderately, severely or completely disabled) or having a TESS or QOL-CS score below the 25th percentile

	**Univariate**			**Multivariate**		
**Subgroups**	**Self-rating disable**	**TESS score**	**QOL-CS score**	**Self-rating disable**	**TESS score**	**QOL-CS score**
⩽12/Amp	1.1 (0.60–1.96)	0.5 (0.26–0.90)*	0.7 (0.40–1.31)	1.5 (0.77–3.10)	0.4 (0.19–0.92)*	1.1 (0.54–2.37)
⩽12/LS	0.7 (0.33–1.56)	0.5 (0.25–1.15)	0.8 (0.40–1.68)	0.9 (0.37–2.19)	0.6 (0.25–1.45)	0.9 (0.37–2.06)
>12/Amp	1.5 (0.90–2.49)	1.0 (0.63–1.67)	1.2 (0.72–1.94)	1.1 (0.74–1.78)	0.9 (0.55–1.59)	1.2 (0.69–2.25)
>12/LS	1.0 (Ref)	1.0 (Ref)	1.0 (Ref)	1.0 (Ref)	1.0 (Ref)	1.0 (Ref)
Poor health	5.4 (2.90–9.93)**	4.1 (2.27–7.59)**	8.3 (4.35–15.89)**	4.9 (2.49–9.76)**	2.8 (1.41–5.47)**	7.5 (3.69–15.24)**
Good health	1.0 (ref)	1.0 (ref)	1.0 (Ref)	1.0 (Ref)	1.0 (Ref)	1.0 (Ref)
Female	1.1 (0.72–1.59)	1.7 (1.17–2.63)*	1.5 (0.98–2.16)	1.1 (0.74–1.78)	1.7 (1.08–2.68)*	1.6 (1.05–2.59)*
Male	1.0 (Ref)	1.0 (Ref)	1.0 (Ref)	1.0 (Ref)	1.0 (Ref)	1.0 (Ref)
						
*Current age (years)*						
⩽30	0.3 (0.19–0.60)**	0.4 (0.20–0.68)*	0.5 (0.26–0.85)*	0.3 (0.13–0.62)**	0.5 (0.22–1.06)	0.5 (0.22–1.03)
31–35	0.5 (0.29–0.84)*	0.6 (0.35–1.00)	0.7 (0.38–1.13)	0.5 (0.26–0.86)*	0.6 (0.33–1.12)	0.7 (0.38–1.34)
36–39	0.6 (0.34–1.0)*	0.7 (0.44–1.29)	1.0 (0.57–1.68)	0.5 (0.28–0.95)*	0.8 (0.42–1.39)	1.0 (0.54–1.79)
⩾40	1.0 (Ref)	1.0 (Ref)	1.0 (Ref)	1.0 (Ref)	1.0 (Ref)	1.0 (Ref)
No high school	5.0 (1.99–2.50)**	4.1 (12.65–10.24)**	2.7 (1.09–6.63)*	4.3 (1.52–12.24)**	4.2 (1.52–12.24)**	1.5 (0.51–4.36)
High school graduate	1.4 (0.92–2.15)	1.2 (0.76–1.81)	0.9 (0.60–1.38)	1.4 (0.88–2.19)	1.2 (0.77–1.96)	0.6 (0.53–1.36)
College graduate	1.0 (Ref)	1.0 (Ref)	1.0 (Ref)	1.0 (Ref)	1.0 (Ref)	1.0 (Ref)
Ewing's sarcoma	0.7 (0.43–1.20)	0.9 (0.54–1.49)	0.8 (0.47–1.29)	—	—	—
Osteosarcoma	1.0 (Ref)	1.0 (Ref)	1.0 (Ref)	—	—	—
Pelvic site	1.3 (0.67–2.52)	1.6 (0.82–3.03)	2.0 (1.08–3.80)*	—	—	0.9 (0.25–3.56)
Limb site	1.0 (Ref)	1.0 (Ref)	1.0 (Ref)	—	—	1.0 (Ref)
Pelvic irradiation	1.3 (0.60–2.61)	1.6 (0.76–3.18)	2.4 (1.22–4.76)*	—	—	2.6 (0.63–10.87)
No pelvic irradiation	1.0 (Ref)	1.0 (Ref)	1.0 (Ref)	—	—	1.0 (Ref)
Limb irradiation	1.1 (0.58–1.94)	0.7 (0.42–1.33)	1.6 (0.82–3.10)	—	—	—
No limb irradiation	1.0 (Ref)	1.0 (Ref)	1.0 (Ref)	—	—	—
Ampution	1.5 (0.97–2.27)	1.0 (0.64–1.47)	1.1 (0.71–1.61)	—	—	—
Limb sparing surgery	1.0 (Ref)	1.0 (Ref)	1.0 (Ref)	—	—	—
⩽12 years old at surgery	0.7 (0.48–1.2)	0.5 (0.31–0.78)**	0.7 (0.44–1.04)	—	—	—
>12 years old at surgery	1.0 (Ref)	1.0 (Ref)	1.0 (Ref)	—	—	—

* *P*<0.05,

** *P*<0.01.

). Those in the ⩽12/Amp group were significantly less likely to score below the 25th percentile of TESS scores compared to subjects in other age/surgery groups, while females were more likely to score below the 25th percentile of TESS scores compared to the males. Those with a pelvic lesion and those receiving pelvic irradiation were more likely to score below the 25th percentile of QOL-CS scores (Table 4).

In multivariable analysis (Table 4) of self-perceived disability, age/surgery group was not predictive; however, poor health status, older current age (>40 years), and not graduating high school predicted an increased likelihood of perceived disability. Those in the ⩽12/Amp group were significantly less likely to score below the 25th percentile in TESS scores. Poor health status, female gender, and not graduating from high school were predictive of TESS scores below the 25th percentile. When examining QOL-CS scores, only female gender and poor health status were predictive of scoring below the 25th percentile.

### Tumour location/surgical procedure

Primary tumour site was not predictive of QOL-CS or TESS scores below the 25th percentile; however, those with a proximal tibia lesion appeared less likely to rate themselves as disabled (see Table 5

**Table 5:** Odds ratio of disability, TESS score (below 25th percentile), or QOL-CS score (below 25th percentile)

	**Self-rating disabled OR (CI)**	**TESS score OR (CI)**	**QOL-CS score OR (CI)**
*Site*			
Pelvis	0.9 (0.36–2.15)	1.3 (0.56–3.16)	1.3 (0.55–3.14)
Proximal tibia	0.5 (0.25–0.96)*	0.7 (0.39–1.42)	0.7 (0.38–1.44)
Distal femur	1.0 (Ref)	1.0 (Ref)	1.0 (Ref)
			
*Surgery*			
Amputation	1.3 (0.82–2.08)	0.8 (0.54–1.34)	1.0 (0.66–1.64)
Limb-sparing	1.0 (Ref)	1.0 (Ref)	1.0 (Ref)

Comparisons by site adjusted for general health status, amputation, education, gender and age. Comparisons by surgical procedure adjusted for general health status, gender, education and age.

* *P*<0.05.

). There was a slightly lower than expected proportion of cases with pelvic tumours (46/528) which typically would account for 25% of Ewing's sarcoma and 10% of osteosarcoma presentations (Arndt and Crist, 1999). This is likely due to the poorer prognosis of those with pelvic tumours at the time this cohort was diagnosed. Analysis of the two surgical groups (amputation *vs* nonamputation) revealed no differences in self-perception of disability or in scoring below the 25th percentile on TESS or QOL-CS after adjusting for general health status, gender, education, and age at questionnaire completion. In the site and surgery group analyses, poor general health and older age at questionnaire completion predicted worse outcomes (data not shown). Female gender and less than a high school education were less consistent in predicting poorer outcomes in the surgical group analysis.

## DISCUSSION

As cure rates for childhood cancers continue to rise, increasing numbers of childhood cancer survivors will be entering adulthood and will require close follow-up for late effects of their therapy. One particular group warranting close evaluation are survivors of bone tumours because of the nature of treatment, which often includes intensive chemotherapy, surgery and, at times, radiation therapy. As reviewed by Langeveld, several studies have already highlighted problems encountered by adult survivors of paediatric bone tumours (Langeveld *et al*, 2002). Late effects relating to chemotherapy and irradiation (e.g. infertility, cardiomyopathy) are varied and have been the subject of extensive research (Bhatia *et al*, 2003). Late effects relating to surgical local control of paediatric lower extremity bone tumours have been less thoroughly explored (Nagarajan *et al*, 2002). Local control procedures include amputation and limb-sparing procedures (e.g. the use of radiation, arthrodesis, endoprosthesis, allograft bone, etc.). The decision to perform a limb-sparing surgery is based on the ability to achieve an equivalent oncologic result and a comparable or better functional result compared to amputation (Yaw, 1999).

Amputation was standard treatment prior to the development of radiological and surgical techniques that have made limb-sparing surgery the more frequent treatment choice (Provisor *et al*, 1997; Bacci *et al*, 1998; Bielack *et al*, 2002). The preference for limb-sparing surgery to treat paediatric lower extremity bone tumours had been the subject of debate and warranted careful evaluation in the past. The issues surrounding the debate have been succinctly described in four questions (Simon, 1991): (1) will survival be the same? (2) how do the immediate and delayed morbidities (complications) compare? (3) how does function compare? and (4) does limb-sparing surgery impart improved psychosocial/quality of life outcomes? Regarding the first question, survival and local recurrence rates between amputation and limb-sparing surgery have not been found to be significantly different when adequate margins are achieved and adjuvant chemotherapy is used (Simon *et al*, 1986; Sluga *et al*, 1999; Bacci *et al*, 2000; Bielack *et al*, 2002). When considering short- and long-term complications, several studies have shown that there are more complications following limb-sparing surgery (Ruggieri *et al*, 1993; Rougraff *et al*, 1994; Lindner *et al*, 1999; Nagarajan *et al*, 2002). Of note, with new techniques and materials being developed for limb-sparig surgeries, long-term outcomes need to be continually evaluated.

In contrast, the last two questions have not been extensively studied, which is the basis for the current investigations. Overall, a trend toward an improvement of function has been reported for limb-sparing approaches. No differences in quality of life have thus far been shown between amputation and limb-sparing operative procedures. However, it is important to note that despite a number of authors addressing these outcomes, the overall conclusions have limitations because of the differing methodologic approaches and assessment tools used, small sample sizes, short follow-up, and limited study of adult survivors of childhood cancer (Nagarajan *et al*, 2002). The most common instrument that has been used for functional assessment has been the Musculoskeletal Tumour Society survey, which relies on the subjective ratings given by the administering clinician with no other objective measure of function. This has been questioned as to whether this is an accurate reflection of function (Marchese *et al*, 2004) and whether other more global functional assessments with QOL measures (TESS and QOL-CS) are needed to better represent the clinical status.

Children with lower extremely bone tumours have unique characteristics with implications for functional and quality-of-life outcomes. The emotional maturity of patients at the time of diagnosis can influence their ability to accept the loss of limb due to cancer (Kagan, 1976; Ettinger and Heiney, 1993; Felder-Puig *et al*, 1998). Skeletal maturity is another important determinant of functional and QOL outcomes in children with lower extremity bone sarcomas because of its importance in determining the type of local surgical control (amputation/rotationplasty/expanding prosthesis) and the associated risk of complications. Those who are skeletally immature and have substantial growth potential at diagnosis are often treated with limb-sparing surgery, which requires removal of a skeletal growth plate. Such patients often need multiple subsequent surgeries to accommodate growth of the unaffected limb. Moreover, children have a substantial lifespan ahead of them following successful treatment of their malignancy and this may increase the potential risk of further reconstructive procedures and complications.

With the establishment of the CCSS to facilitate the investigation of late effects among long-term survivors of childhood cancer, we were able to examine some of these key issues. The current study represents the largest series of adult survivors of paediatric lower extremity bone tumours thus far evaluated for function and QOL. However, some limitations must be considered in the interpretation of the results of this study. These include the inability to further classify the procedures that nonamputees (e.g. allograft *vs*. endoprosthesis) and amputees (above the knee *vs* below the knee amputation) received and the fact that this study provides information regarding treatments performed between 1970 and 1986, which are likely quite different from today's surgical treatments. However, this study does provide an excellent assessment of the outcomes of amputees, which would be difficult to examine today given the infrequency with which amputations are performed for paediatric lower extremity bone tumours. An additional shortcoming of the study relates to the length of time from diagnosis (median 21 years) to current assessment, which precludes the examination of outcomes within 10 years of diagnosis. It is in this time frame that differences between groups may be substantial and may be masked or lost by longer follow-up. Other issues involved the assessment of subsequent surgeries or complications. Since the assessment of surgeries was restricted to the initial treating institution and complications were not specifically noted, the accurate enumeration of subsequent surgeries and complications is not possible. Further limitation of the study includes ascertainment bias since the cohort includes only those who have agreed to participate in the CCSS. This may underestimate deficits by excluding those who are having more difficulty adjusting and are unable or unwilling to participate.

We found no major differences in function and quality of life between those who had an amputation and those treated with limb-sparing surgery. Additionally, our assessment of the large population of patients who underwent amputation provides clear indication that amputees do well long-term. Thus, when an amputation is clinically indicated, patients, families and clincians can assume that long-term amputees have no different function and QOL compared to those without an amputation. One may attribute the lack of reported differences between amputees and nonamputees to ‘adjustment and accommodation’ to their current condition, rather than having comparable physical ability such as range of motion and strength. To determine actual physical ability, via clinical examination, in a cohort as large as this and as far from diagnosis would be very difficult to accomplish. Additionally, one may argue that ‘actual’ physical ability is only a component of overall perceived physical function, which is influenced by other individualised factors including self-image, motivation and social support (Neugebauer, 2000).

Females reported significantly lower function and QOL, but did not report more disability. Survivors with a lower educational attainment also appeared to have lower function and QOL scores and a significantly increased likelihood of self-reported disability. In the author's prior study of psychosocial outcomes (Nagarajan *et al*, 2003) in this group of survivors, female survivors were less likely to be employed and those with a higher educational attainment were more likely to ever been employed or married or ever have insurance. Female gender-related deficiencies may be related to differences in coping styles (Znajda *et al*, 1999) and other gender-specific issues and the benefit of higher educational attainment may suggest more available opportunities or better social support. In the current study, those who were younger in age at completion of the questionnaire were less likely to have lower TESS and QOL-CS scores and significantly less likely to consider themselves disabled. This is most likely due to issues related to normal aging.

Further follow-up of this cohort, including reassessment to observe any changes in scores and perceived disability over time, is clearly warranted. It will also be important to prospectively investigate post-1986 populations of paediatric lower extremity bone tumour survivors in order to see how current surgical techniques affect function and quality of life. These prospective studies must encompass long-term assessments and integrate uniform methods of recording complications associated with the surgical procedures, as well as uniform collection of function and QOL data. Such an approach will aid paediatric oncologists and orthopaedic surgeons by providing insight into disease control, functional outcomes, and quality of life. We hope that this research study will help provide patients and families with needed information on anticipated long-term outcomes and on how to maximise function and quality of life as these paediatric patients grow into adulthood.

## Appendix

### CCSS Institutions and Investigators

**Table A1:**

University of California, San Francisco, CA	Arthur Ablin, MD*
University of Alabama, Birmingham, AL	Roger Berkow, MD*
International Epidemiology Institute, Rockville, MD	John Boice, ScD‡
University of Washington, Seattle, WA	Norman Breslow, PhD‡
UT-Southwestern Medican Center at Dallas, TX	George R, Buchanan, MD*, Kevin Oeffinger. MD‡
Cincinnati Children's Hospital Medical Center	Stella Davies, MD, PhD‡
Dana-Farber Cancer Institute, Boston, MA	Lisa Diller, MD*, Holcombe Grier, MD†, Frederick Li MD‡
Texas Children's Center, Houston, TX	Zoann Dreyer, MD*
Children's Hospital and Medical Center, Seattle, WA	Debra Friedman, MD, MPH*, Thomas Pendergrass, MD†
Reswell Park Cancer Institute, Buffalo, NY	Daniel M Green, MD*‡
Hospital for Sick Children, Toronto, NY	Mark Greenberg, MB ChB*
St. Louis Children ‘s Hospital, MO	Robert Hayashi, MD*, Teresa Vietti, MD†
St. Jude Children's Research Hospital, Memphis, TN	Melissa Hudson MD*‡
University of Michigan, Ann Arbor, MI	Raymond Hutchinson MD*
Stanford University School of Medicine, Stanford, CA	Michael P Link, MD*, Sarah S Donaldson, MD‡
Emory University, Atlanta, GA	Lillian Meacham, MD*
Children's Hospital of Philadelphia PA	Anna Meadows, MD*‡
Children's Hospital, Oklahoma City, OK	John Mulvihill MD‡
Children's Hospital, Denver, CO	Brain Greffe*, Lorrie Odom, MD†
Children's Health Care-Minneapolis, MN	Maura O'Leary, MD*
Columbus Children's Hospital. OH	Amanda Termuhlen MD*, Frederick Ruymann, MD†, Stephen Qualman, MD‡
Children's National Medical Center. Washington, DC	Gregory Reaman, MD*, Roger Packer, MD‡
Children's Hospital of Pittsburgh, PA	A Kim Ritchey, MD*, Julie Blatt MD†
University of Minnesota. Minneapolis. MN	Leslie L. Robison PhD*‡, Ann Mertens, PhD‡, Joseph Neglia, MD, MPH‡, Mark Nesbit, MD‡,
Children's Hospital Los Angeles, CA	Kathy Ruccione, RN, MPH*
Memorial Sloan-Kettering Cancer Center New York	Charles Sklar, MD*‡
National Cancer Institute, Bethesda, MD	Malcolm Smith, MD‡, Peter Inskip, ScD‡
Mayo Clinic, Rochester, MN	W. Anthony Smithson, MD*, Gerald Gilchrist, MD†
UTMD Anderson Cancer Center, Houston, TX	Louise Strong, MD*‡, Marilyn Stovall, PhD‡
Riley Hospital for Children, Indianapolis IN	Terry A, Vik, MD*‡, Robert Weetman, MD‡
Fred Hutchinson Cancer Center, Seattle. WA	Yutaka Yasui, PhD*‡, John Potter, MD., PhD*‡
University of California-Los Angeles. CA	Lonnie Zeltzer, MD*‡

* Institutional Principal Investigator

† Former Institutional Principal Investigator

‡ Member CCSS Steering Committee
